# Increased expression of OPN contributes to idiopathic pulmonary fibrosis and indicates a poor prognosis

**DOI:** 10.1186/s12967-023-04279-0

**Published:** 2023-09-19

**Authors:** Jie Ji, Shudan Zheng, Yuxin Liu, Tian Xie, Xiaoyu Zhu, Yang Nie, Yi Shen, Xiaodong Han

**Affiliations:** 1 Immunology and Reproduction Biology Laboratory and State Key Laboratory of Analytical Chemistry for Life Science, Medical School, Medical College of Nanjing University, Hankou Road 22, Nanjing, 210093 China; 2 Jiangsu Key Laboratory of Molecular Medicine, Nanjing University, Nanjing, 210093 China; 3 Department of Cardiothoracic Surgery, Jinling Hospital, Medical School of Nanjing University, Nanjing, China

**Keywords:** Idiopathic pulmonary fibrosis (IPF), Prognostic, Osteopontin (OPN), Alveolar epithelial cell, Epithelial-mesenchymal transition (EMT)

## Abstract

**Background:**

Idiopathic pulmonary fibrosis (IPF) is fibrotic lung disease with no effective treatment. It is characterized by destruction of alveolar structure and pulmonary interstitial fibrosis, leading to dyspnea and even asphyxia death of patients. Epithelial-mesenchymal transition (EMT) is considered to be a driving factor in the pathogenesis of IPF. Osteopontin (OPN) is a secreted protein widely present in the extracellular matrix and involved in the occurrence and development of a variety of diseases.

**Methods:**

The original datasets were obtained from NCBI GEO databases analyzed with the online tool GEO2R and EasyGEO. Bleomycin induced mouse pulmonary fibrosis model and OPN/OPN-biotin treated mouse model were established to investigate the role of OPN in mouse pulmonary fibrosis and the target cells of OPN. A549 cells and HBE cells were used to explore the mechanism of OPN-induced epithelial-mesenchymal transition (EMT) in epithelial cells and mass spectrometry was used to detect OPN downstream receptors. Precision-cut lung slices and lentivirus-treated mice with pulmonary fibrosis were used to examine the therapeutic effect of OPN and its downstream pathways on pulmonary fibrosis.

**Results:**

We demonstrate that the content of OPN in IPF bronchoalveolar lavage fluid (BALF) is high compared to the normal groups, and its expression level is correlated with prognosis. At the animal level, OPN was highly expressed at all stages of pulmonary fibrosis in mice, and the bronchoalveolar lavage fluid (BALF) could accurately reflect its expression in the lung. Next, we reveal that OPN was mainly expressed by macrophages and the main target cells of OPN were epithelial cells. Mice developed pulmonary fibrosis accompanied after treating the mice with OPN. Both in vitro and in vivo experiments confirmed that OPN could induce EMT of alveolar epithelial cells. Mechanistically, OPN binding triggered phosphorylation of FAK by CD44, thus activating snail1-mediated profibrotic protein synthesis. Inhibition of FAK phosphorylation and its downstream pathways can effectively alleviate pulmonary fibrosis in precision sections of lung tissue (PCLS) assay. OPN knockdown in bleomycin-induced lung fibrosis mice led to significantly less fibrosis.

**Conclusion:**

Our data suggest that OPN mediates lung fibrosis through EMT, implicating its potential therapeutic target and prognostic indicator role for IPF. OPN may be a target for the diagnosis and treatment of IPF.

**Supplementary Information:**

The online version contains supplementary material available at 10.1186/s12967-023-04279-0.

## Introduction

Idiopathic pulmonary fibrosis (IPF) is a progressive and fibrotic interstitial lung disease associated with unknown etiology and worse prognosis [1]. The effect of existing treatment strategies is unsatisfactory and has side effects [2]. Even worse, the morbidity and mortality of IPF are increasing year by year [3]. Therefore, new target molecules and therapeutic regimens are urgently needed.

Multiple pathogenesis has been theorized to initiate and maintain lung fibrosis, such as repetitive alveolar epithelial injury, fibroblast activation, macrophage polarization and so on [4, 5]. In the above, AT2 cell injury is recently recognized as IPF driver [6]. AT2 cell is able to transdifferentiate into AT1 cells and self-renew, contributing to the production of pulmonary surfactant [7]. Alveolar epithelial injury includes epithelial-mesenchymal transition (EMT), cell death, cell senescence and other disorders, leading to the disruption of the epithelial barrier, impair gas exchange and further fibrosis [6]. Several risk factors have been involved in the causes of alveolar epithelial injury, including external environment and intrinsic factor [8]. Of these, the intercellular interaction is the most complex due to the complicated network of cells. In previous studies, AT2/mesenchymal crosstalk and immune-epithelial crosstalk were found to amplify the initial injury and fibrosis [9]. This reminds us that cell crosstalk and their secretory protein may play a vital role in IPF pathology.

Osteopontin (OPN) is a highly phosphorylated glycophosphoprotein, which is encoded by the secreted phosphoprotein 1 (SPP1) gene [10]. OPN exists extensively in a large number of tissues such as kidney, brain, bone marrow and lung [11]. OPN is a "soluble" extracellular matrix (ECM) molecule that exists extensively in a large number of tissues such as kidney, brain, bone marrow and lung [12]. Physiologically, it is believed that OPN expression is high in the most case of tissues and body fluids that is involved in wound healing and remodeling [13]. Moreover, the OPN expression is detected in multiple pathological conditions due to its function of regulating cell migration and differentiation [14]. For example, serum and hepatic OPN levels are increased in patients with alcohol-associated liver disease (ALD) and nonalcoholic fatty liver disease (NAFLD) [11]. In terms of lung disease, OPN is found to facilitate lung cancer development by promote cell epithelial-to-mesenchymal transitions and proliferation [15]. Furthermore, a study shows that serum OPN could predict the survival in IPF patient [16]. In our previous studies, we found that alternative macrophage activation is dependent on OPN expression, thus contributing to the development of pulmonary fibrosis [17]. According to the above research results, OPN may participates in the pathological processes of IPF. we designed experiments to investigate the role of OPN in the development of IPF.

Thus, we sought to identify the role of OPN on the development of IPF. We found elevated SPP1 in the bronchoalveolar lavage fluid (BALF) from IPF in GSE70867, and the expression level is associated with the prognosis of IPF. Meanwhile, OPN expression increases in fibrotic lung tissue of mice, which supporting a role for OPN in IPF development. Furthermore, abnormally high expression of OPN is found mainly in macrophages of fibrotic lung tissues. The enhanced expression of OPN exerted the profibrotic role by promoting alveolar epithelial cells EMT. Mechanistically, OPN combined with CD44 and triggered FAK phosphorylation that further phosphorylates AKT, contributing to the expression of snail1 and downstream profibrotic genes. Our findings present the OPN/CD44/P-FAK/signal cascade as a therapeutic target for the treatment of IPF.

## Methods and materials

### Data acquisition and processing

The original datasets were obtained from NCBI GEO databases. We selected three datasets, GSE70866 [18], GSE70867 [18] and GSE28042 [19, 20] and analyzed with the online tool GEO2R and EasyGEO. Among the two datasets, GSE70866 and GSE70867 were generated from Germany, while the other was from USA. GSE70866 contains sequencing data of BAL cells from 212 IPF patient and 20 healthy donors. The results identify and validate a BAL cell gene expression signature that predicts mortality in IPF. In our research, we divided the samples in GSE70866 into two groups, namely control and IPF. The differential genes of GSE70866 were analyzed accordingly, cut-off criteria were set at p-value < 0.05 and logFC (> 1 or < 0.05) was considered as statistically significant. Furthermore, the specific difference of OPN expression between the control group and the IPF group in GSE70866 was compared. At the same time, in addition to the specific amount of OPN expression in dataset GSE70866, the dataset GSE70867 and GSE28042 also contain clinical information such as survival state, survival time and age of IPF patients. GSE28042 containns microarray analyses of PBMC from 120 patients, while GSE70867 contains gene expression and clinical information of alveolar lavage in 212 patients with IPF. We used EasyGEO to obtain the clinical survival information of GSE70867 and GSE28042 samples. We first performed a linear correlation analysis of the survival days of samples and the corresponding OPN expression levels. Next, the receiver operating characteristic (ROC) curve was used for evaluating model discrimination. We regrouped the samples according to the OPN expression level, and those above the median were defined as OPN-high group, and those below the median were defined as OPN-low group. On this basis, we compared the survival of IPF patients in the two groups.

### Reagents

Bleomycin (BLM) was purchased from Nippon Kayaku (Tokyo, Japan). The P-FAK inhibitor defactinib (a specific FAK phosphorylation inhibitor) were purchased from Medchemexpress (no. HY-12289). Mouse and human recombinant OPN were purchased from R&D Systems (no. 441-OP-050, no. 1433-OP-050). Mouse biotin-OPN was synthesized by Medchemexpress. The LV-OPN-siRNA was synthesized by GENECHEM (Shanghai, China). Antibodies used in this study were listed in Additional file 3: Table S1.

### Patients

Human lung tissue sections were obtained from lung tissue specimens of Nanjing Drum Tower Hospital. Controls (n = 3) were selected to be similar in age to IPF (n = 5) with no fibrotic lung disorders. Use of samples was approved by the Ethics Committee of Nanjing Drum Tower Hospital.

### Animals and treatment

Mice contributed to the study were male SPF C57BL/6 animals. All animal procedures were approved by the Ethics Committee for Animal Research of Medical School of Nanjing University.

#### Bleomycin induced mouse pulmonary fibrosis model

To establish pulmonary fibrosis model, mice was injected intratracheally with 50 µl 5 mg/kg bleomycin or saline and sacrificed 7, 14, 21 or 28 days after bleomycin instillation.

#### OPN treated mouse model

For OPN administration, recombinant mouse OPN or biotin-OPN in 50 µl of PBS (50 mg/kg) was instilled into the trachea every 3 days for one month.

### Elisa

Bronchoalveolar lavage fluid (BALF) from mice was collected for ELISA, and the specific procedures were referred to previous studies in our laboratory. In brief, mouse alveolar lavage fluid was centrifuged at 1000 g for 5 min, and the supernatant was taken, diluted 5 times, and detected using a kit. The final content was determined from the standard curve in the kit.

### Lung hydroxyproline assay in mouse lung samples

The tissue wet weight of 50 mg was accurately weighed into the test tube, accurate Add 1 mL of hydrolysate and mix well. After capping, 95 °C or boiling the water bath was hydrolyzed for 20 min. After cooling the running water of each test tube, 10 μl of indicator was added to each tube and shaken well. The pH value was adjusted to about 6.0–6.8. The supernatant was separated at 3500 RPM for 10 min, and then detected at a wavelength of 550 nm.

### Histology, immunohistochemistry and immunofluorescence staining

The specific steps of embedding and sectioning experiments of mouse lung tissues were carried out according to previous studies, as were HE and MASSON experiments. The immunohistochemistry staining of lung tissues immunofluorescence analysis of A549, HBE cells or lung tissues were performed as described previously. The secondary antibodies incubated were horseradish peroxidase-conjugated goat anti-mouse IgG for immunohistochemistry, Alexa Fluor 488-conjugated goat anti-rabbit antibody or Alexa Fluor 594-conjugated goat anti-mouse antibody (Invitrogen) for Immunofluorescence. Besides, nuclei were stained with DAPI (Sigma) for Immunofluorescence.

### Cell culture

A549 cells and HBE cells were obtained from the American Type Culture Collection (ATCC, Manassas, VA, USA) and cultured in DMEM (Wisent) or 1640 supplemented with 10% FBS and 1% penicillin and streptomycin at 37 °C in 5% CO_2_. A549 cells and HBE cells were treated with OPN (1 μg/ml) or not, and harvested for RNA and protein extraction, immunofluorescence scratch test and migration test after 48 h.

To silence CD44 expression, A549 cells were transfectedwith LV-CD44-siRNA according to the lentiviral protocolsprovided by GENECHEM.

### Scratch test and migration test

A549 cells were seeded in 6-well plates after treated with OPN for 48 h. The confluent monolayers were wounded as previously described [21]. Images were captured immediately at 0 h and 48 h. Images were blindly analysed for cell migration ability by ImageJ.

### Precision-cut lung slices (PCLS)

Selected lobes of mouse lungs were isolated and expanded with 1.5% low-gelling agarose (Sigma Aldrich) in the culture medium. Expanded agarose lobes were placed on dry ice for 30 min to allow the agarose to solidify, and then cut into 1-cm-thick slices. Tissue cores were prepared from 1-cm-thick sections using an 8-mm coring tool (Alabama Research and Development Munford AL). Cores were sectioned at a thickness of approximately 500-um using a Krumdieck tissue microtome MD6000(Alabama Research and Development) containing cold phosphate buffered saline (PBS). Lung sections were cultured in a humidified incubator with 5% CO2 95% air at 37 °C. The lung slices were washed every 30 min for a total of four times and incubation medium was used. Lung sections were placed in culture medium and incubated in 12-well culture plates at 37 °C using three to four sections per well. The next day, sections were placed into 24-well tissue culture plates, one slice per well, in 1 ml of medium and maintained under tissue culture conditions. The medium was changed daily.

### Quantitative real-time polymerase chain reaction (qPCR)

For analysis of mRNA, HiScript 1st strand cDNA Synthesis Kit (Vazyme Biotech Co, Nanjing, China) was used for RT-PCR reaction. Specific primers for mRNAs are listed in Additional file 4: Table S2.

### Western blotting and co-immunoprecipitation

Cells or lung tissues were lysed by RIPA buffer to purify proteins. Western blot analysis was performed as previously described [22]. Horseradish peroxidase-conjugated IgG was used as a secondary antibody. Co-IP was performed using the Thermo Scientific Pierce Co-IP kit following the manufacture’s protocol.

### Statistics

Statistical analysis was carried by GraphPad Prism 8.0.1. Differences between groups were calculated by one-way ANOVA analysis followed by tukey's multiple comparisons test. The data are presented as means ± SD and the results are considered statistically significant if p < 0.05.

## Results

### OPN expression is increased in IPF lung and that OPN expression is an indicator of poor prognosis in IPF

**Fig. 1:**
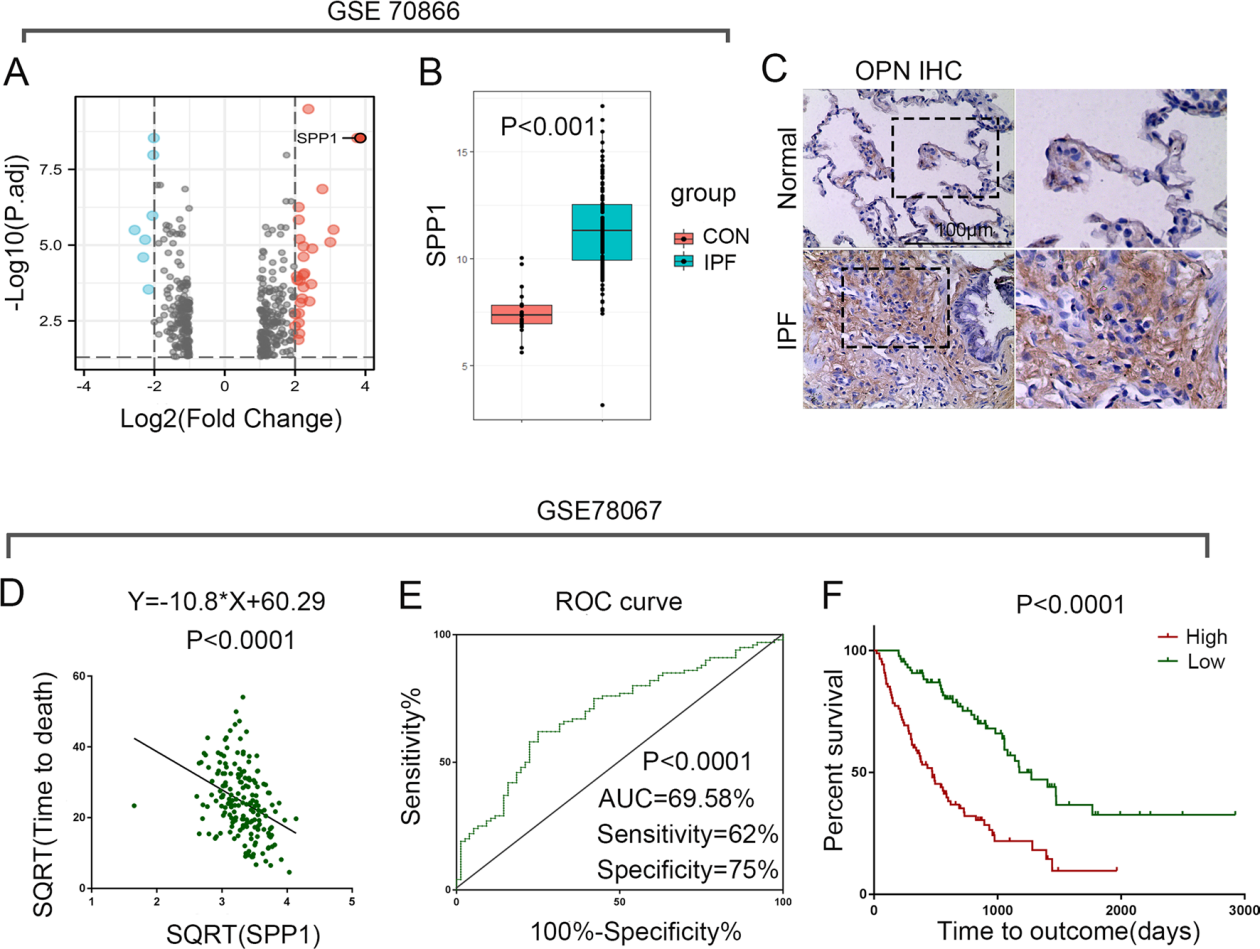
OPN is highly expressed in the lung tissue of IPF patients and is associated with the prognosis of patients. **A** Volcano plot visualizing the DEGs in GSE70866. The x axis indicates the fold change (log-scaled), whereas the y axis shows. Vertical lines represent either upregulation or downregulation by twofold. The horizontal line represents a p-value = 0.05. Red points represent upregulated (up) genes, gray points stand for no-significantly-changed (NSC) genes, and blue points represent downregulated genes. **B** Box plot shows the difference of the level of OPN expression between control group and IPF groups in GSE70866, p < 0.0001. **C** The lung biopsy tissue sections of IPF and donor were subjected to immunohistochemical staining of OPN. Bar, 100 μm. **D** The survival time of IPF patients was linearly correlated with the expression level of OPN in GSE70867, p < 0.0001. **E** ROC curves for forecasting overall survival, p < 0.0001. **F** Kaplan–Meier plot of overall survival between OPN high and OPN low groups in GSE70867

In order to assess the impact of OPN on the prognosis and development of IPF, we reanalyzed the gene expression profile of GSE70866, founding that the expression level of IPF alveolar lavage fluid is times higher than control groups (Fig. 1A, B). As a complement, we examined OPN expression in sections obtained from IPF patients and controls. This suggests that OPN expression is also higher in IPF lung tissues than in controls (Fig. 1C).

In order to explore the relationship between the expression level of OPN and the survival time of patients, we fitted the correlation with the expression level of OPN as the independent variable and the survival time of patients as the dependent variable, and found that the two were negatively correlated in GSE70867 (Fig. 1D). Furthermore, ROC curve was performed on dataset GSE70867 and GSE28042 (Fig. 1E, Additional file 1: Fig. S1B). It was found that in the dataset GSE70867, the expression of OPN had a good predictive effect on the survival status of patients, with the expression of AUC = 69.58%, P < 0.0001 (Fig. 1E). However, in dataset GSE28042, the expression level of OPN was not ideal for predicting the survival status of patients (Additional file 1: Fig. S1B). Based on the above results, patients were further grouped according to the median OPN expression level, and the difference in survival curves between the two groups was compared. Consistent with the above results, the analysis found that in the dataset GSE70867, the survival rate of the group with high OPN expression was lower and statistically significant (Fig. 1F), while in dataset GSE28042, there was no significant difference in survival between the two groups (Additional file 1: Fig. S1C). These results indicated that the content of OPN in lung tissue and alveolar lavage fluid of IPF patients was significantly higher than that of control group. Moreover, high levels of OPN in alveolar lavage fluid rather than peripheral blood suggested poor prognosis in patients with IPF (Additional file 2: Fig. S2).

### OPN expression increases in fibrotic lung tissue of mice

**Fig. 2:**
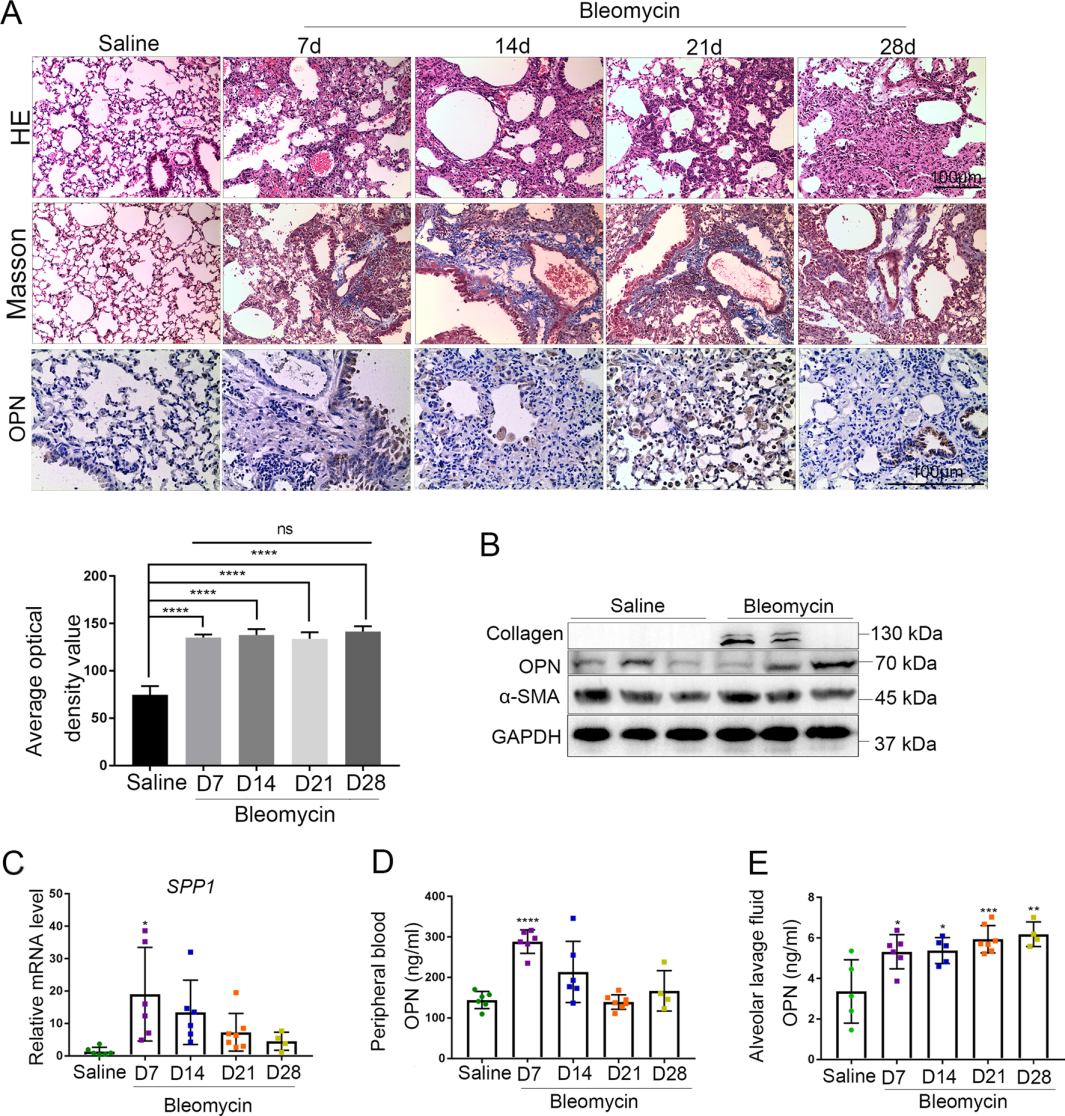
OPN expression increases in the lung of bleomycin-treated mice. **A**–**E** Bleomycin (5 mg/kg body weight) or saline were administered daily in mice (n≧4), and parameters analyzed on day 7, 14, 21 and 28. **A** Representative H&E, Masson and IHC staining of mouse lung tissue. Scale bar, 100 μm. Each group was given 5 different visual fields and the positive areas were quantified by average optical density value (n = 3), ****P < 0.0001, ns denotes no significance compared with day 7 group; means + SD. **B** Immunoblots of OPN, collagen and α-SMA of mouse lung tissue. **C** QPCR analysis of OPN gene (spp1) expression in the lung of bleomycin-treated mice. 18S was used as the reference gene, *P < 0.05; means + SD. **D** Concentration of OPN in peripheral blood determined by ELISA, ****P < 0.0001; means + SD. **E** Concentration of OPN in bronchoalveolar lavage fluid (BALF) determined by ELISA, *P < 0.05, **P < 0.01, ***P < 0.001; means + SD

To assess the pathological stage of fibrosis of the mouse pulmonary fibrosis (PF), we performed H&E and Masson staining in lung sections from bleomycin induced mouse PF (Fig. 2A) and from control groups. And to determine the relevance of OPN to the mouse pulmonary fibrosis (PF), the expression level of OPN in was determined by immumohistochemical staining (Fig. 2A) and western blot (Fig. 2B). The western blot result shows that OPN is upregulated in PF mouse lungs (Fig. 2B) and the IHC staining is consistent with this result (Fig. 2A). Moreover, we observed an increase in OPN mRNA level in PF mouse lung sections compared with the control groups (Fig. 2C), confirming our proteomic analyses. Considering that OPN is a secretory protein, we next tested the concentration of OPN in peripheral blood (Fig. 2D) and bronchoalveolar lavage fluid (Fig. 2E). The results show that the content of OPN in peripheral blood is generally increased in fibrosis group, but only the change in the early stage of fibrosis was significant. Unlike in the peripheral blood, the content of OPN in alveolar lavage fluid increased continuously and significantly.

### OPN is mainly expressed by macrophages

**Fig. 3:**
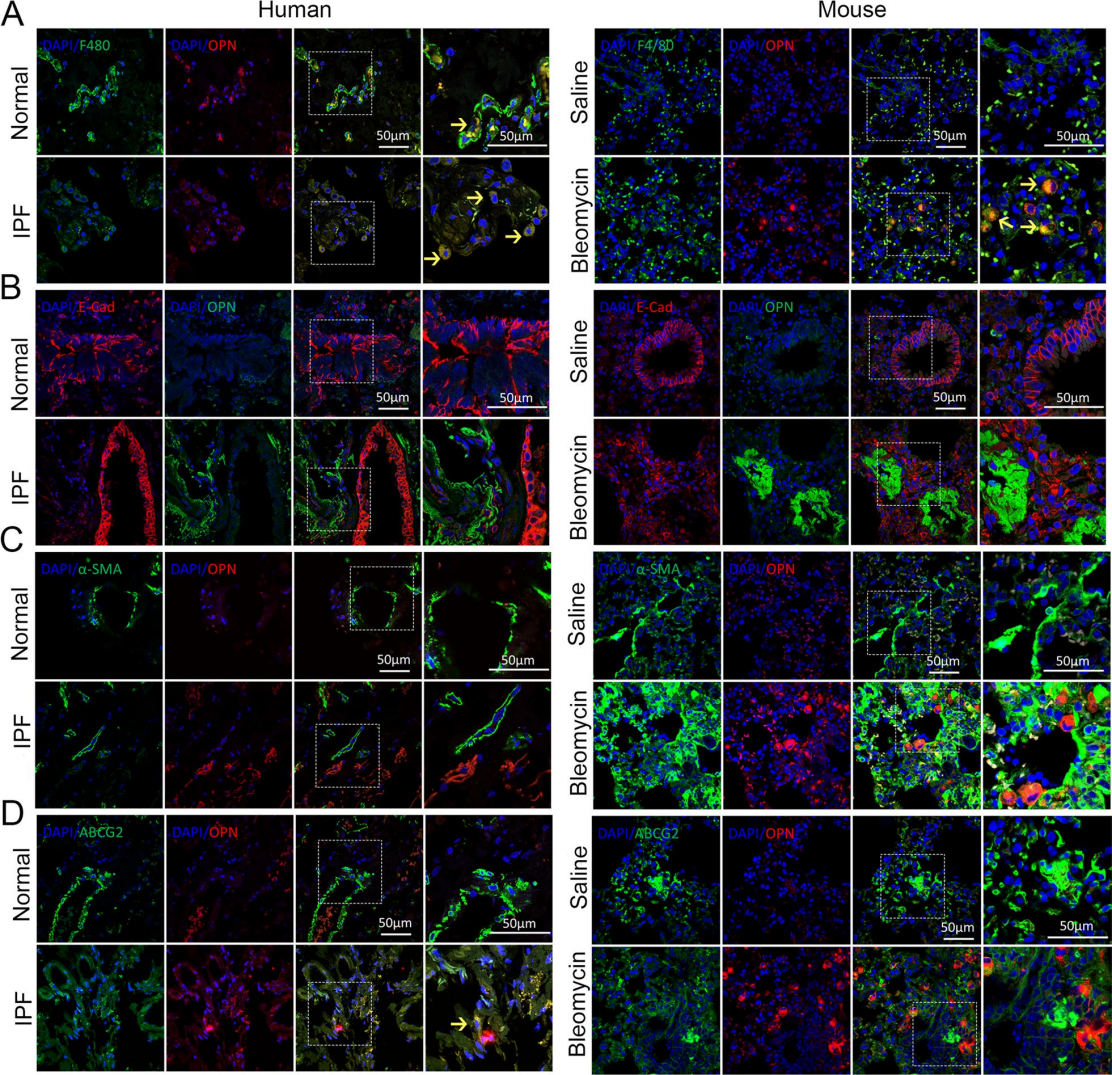
OPN is expressed in macrophages of fibrotic lung tissue. **A** Confocal microscopy showing F4/80 (green), OPN (red), and DNA dye DAPI (cyan) in lung tissue section of IPF and mice treated with bleomycin and matched group, showing the co-localization of OPN and macrophages. Bars, 50 µm. Co-location cells are indicated by yellow arrows. **B** Confocal microscopy showing OPN (green), E-cadherin (red), and DNA dye DAPI (cyan) in lung tissue section of IPF and mice treated with bleomycin and matched group, showing the co-localization of OPN and epithelial cells. Bars, 50 µm. **C** Confocal microscopy showing α-SMA (green), OPN (red), and DNA dye DAPI (cyan) in lung tissue section of IPF and mice treated with bleomycin and matched group, showing the co-localization of OPN and fibroblasts. Bars, 50 µm. **D** Confocal microscopy showing ABCG2 (green), OPN (red), and DNA dye DAPI (cyan) in lung tissue section of IPF and mice treated with bleomycin and matched group, showing the co-localization of OPN and mesenchymal stem cells. Bars, 50 µm

To further clarified the cellular source of OPN during pulmonary fibrogenesis, we performed double immunofluorescence staining of OPN with lung macrophage cell marker F4/80 (Fig. 3A), epithelial cell marker E-cadherin (Fig. 3B), fibroblast cell marker α-SMA (Fig. 3C) and lung-resident mesenchymal stem cell marker ABCG2 (Fig. 3D), respectively. Results showed that OPN staining were predominantly observed in F4/80^+^ macrophages in the lung of IPF and bleomycin-treated mice, co-location cells are indicated by yellow arrows. However, the expression of OPN in other types of cells was not detected. These results suggested that OPN was mainly expressed by macrophages in the fibrotic lung.

### OPN drives pulmonary fibrosis development

**Fig. 4:**
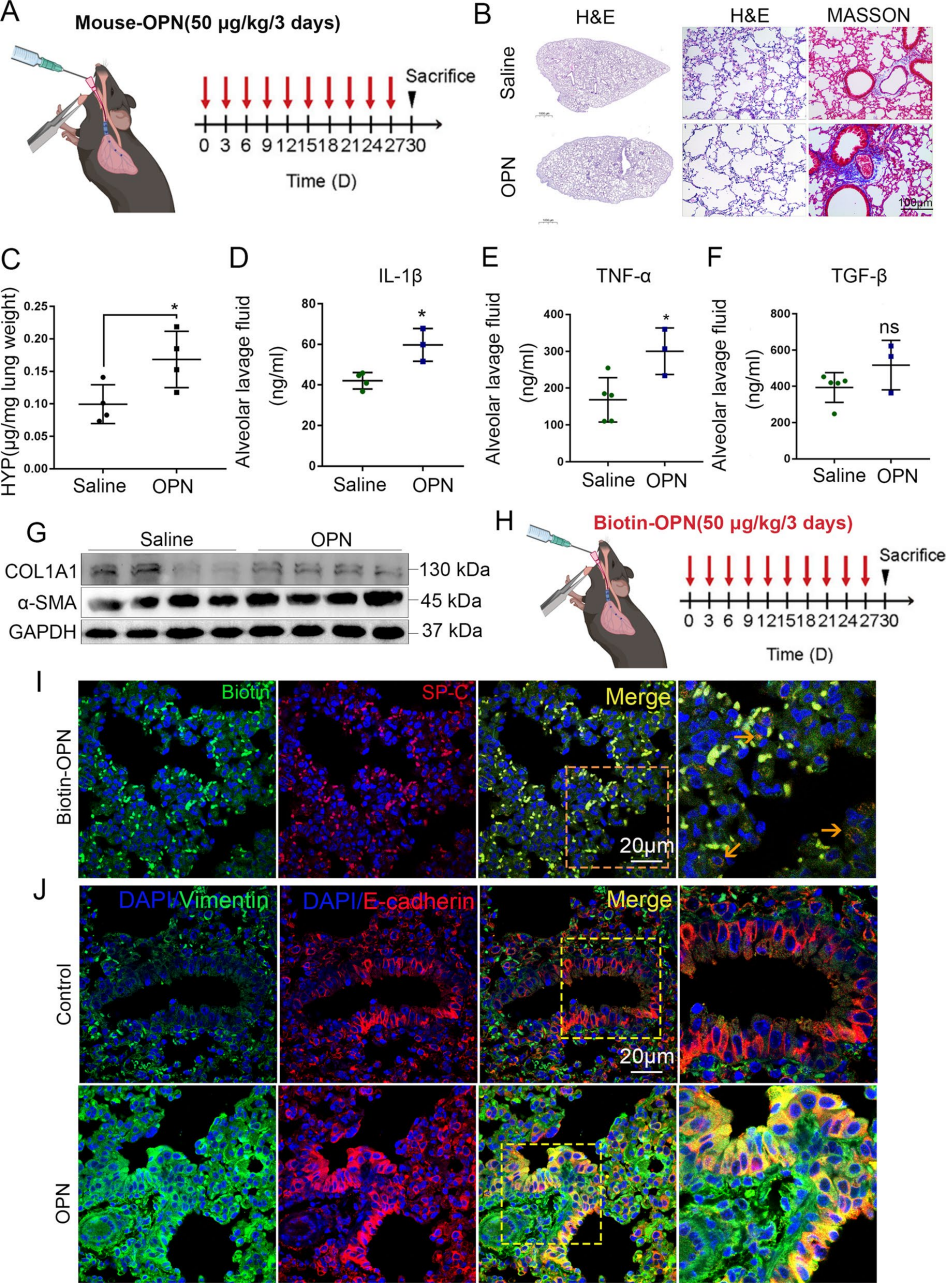
OPN effects alveolar epithelial cell and promotes lung fibrogenesis and the development of PF. **A** Schematic overview of experimental design for (**B**–**G**), recombinant mouse OPN in 50 µl of PBS (50 mg/kg) was instilled into the trachea every 3 days for one month. (n≥4). **B** H&E and Masson staining of lung tissues after multiple OPN exposures. Scale bar, 1000 μm or 100 μm. **C** Lung tissue hydroxyproline content was measured in differently treated animals. *P < 0.05; means + SD. **D**–**F** Concentration of IL-1β (**D**), TNF-α (**E**) and TGF-β (**F**) in bronchoalveolar lavage fluid (BALF) determined by ELISA. *P < 0.05, ns denotes no significance compared with saline group; means + SD. **G** Immunoblots of collagen and α-SMA of mouse lung tissue. **H** Schematic overview of experimental design for (**I**, **J**), recombinant mouse biotin-OPN in 50 µl of PBS (50 mg/kg) was instilled into the trachea every 3 days for one month. (n≥4). **I** IF of streptavidin-Texas (red) with SP-C (green) in biotin-OPN challenged mice. Scale bars, 20 μm. The orange arrows indicate immunofluorescence co-locatable cells. **J** Confocal microscopy showing vimentin (green), E-cadherin (red), and DNA dye DAPI (cyan) in lung tissue section of mice treated with OPN and matched group, showing epithelial-mesenchymal transition. Bars, 20 µm

To determine whether OPN promoted lung fibrogenesis in vivo, mice were repeatedly injected intratracheally with recombinant OPN (Fig. 4A). Exogenous OPN induced fibrotic changes in mouse lungs, as revealed by the damaged alveolar structure and increased collagen deposition (Fig. 4B). Besides, the hydroxyproline content of the lung tissue treated with OPN was higher than the control group, indicating increased collagen synthesis (Fig. 4C). Furthermore, the content of IL-1β, TNF-α in bronchoalveolar lavage fluid (BALF) of OPN groups increased, indicating the occurrence of alveolar inflammation (Fig. 4D, E). The change of TGF-β content in BALF of OPN groups was not significant, suggesting that OPN induced pulmonary fibrosis in mice is different from the classical TGF-β pathway (Fig. 4F). Further experiments confirmed that fibrosis marker protein α-SMA and collagen were upregulated (Fig. 4G). These data suggest that OPN are able to promote fibrosis in lung tissue. We next explored the cellular targets of OPN during lung fibrogenesis (Fig. 4H). Lung tissues from multiple biotin-OPN challenged mice were immunostained for streptavidin-Texas with F4/80, E-cadherin, or a-SMA. We observed elevated OPN levels in E-cadherin^+^ epithelial cells, but not in fibroblasts (Fig. 4I), suggesting that epithelial cells mainly took up OPN. We next focus on the change of epithelial cells. Considering that epithelial cells epithelial-mesenchymal transition (EMT) contributes to pulmonary fibrosis development, we tested the EMT marker in epithelial cells. In line with expectations, we found vimentin was upregulated in epithelial cells of OPN treated mouse, indicating that the EMT occurs (Fig. 4J). The above results show OPN induces pulmonary fibrosis, accompanied by pulmonary inflammation, which may be due to the occurrence of EMT.

#### OPN promotes alveolar epithelial cells epithelial-mesenchymal transition.

**Fig. 5:**
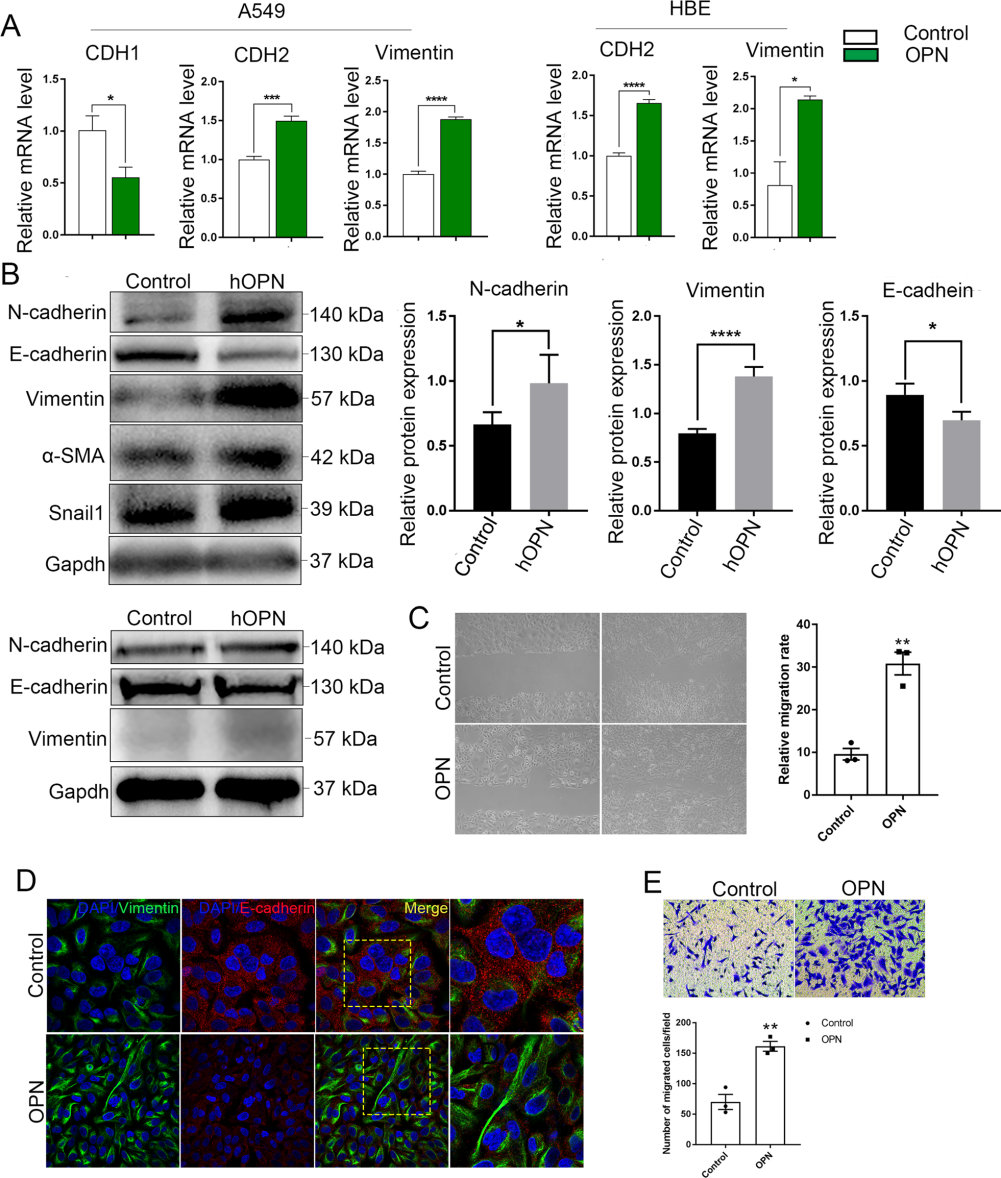
OPN promotes alveolar epithelial cells epithelial-mesenchymal transition in vitro. **A** QPCR analysis of CDH1, CDH2, and vimentin expression in A549 and HBE cell lines. *P < 0.05, **P < 0.01, ***P < 0.001, ****P < 0.0001; means + SD. **B** Immunoblots of N-cadherin, E-cadherin, vimentin, α-SMA and snail1 of A549 cells; immunoblots of N-cadherin, E-cadherin and vimentin of HBE cells. *P < 0.05, ****P < 0.0001; means + SD. **D** Confocal microscopy showing vimentin (green), E-cadherin (red), and DNA dye DAPI (cyan) in A549 cells treated with OPN and matched group, showing epithelial-mesenchymal transition. Bars, 20 µm. **C** The migration capacity of A549 cells was detected by using a wound-healing assay. Wound areas were calculated by ImageJ. **P < 0.01; means + SD. **E** The invasion ability of A549 cells was detected by using transwell assay. The number of cells was calculated by ImageJ. **P < 0.01; means + SD

To further investigate whether OPN can induce EMT in alveolar epithelial cells in vitro, we treated alveolar epithelial cell lines with OPN and examined whether EMT changes occurred. QPCR results show CDH1 was downregulated, CDH2 and vimentin were upregulated in A549 cells. Meanwhile, the mRNA level of CDH2 and vimentin were upregulated in HBE cells (Fig. 5A). Further experiments confirmed that N-cadherin, vimentin, snail1 and α-SMA and collagen were upregulated in A549 cells and E-cadherin is downregulated on protein level, and the same is true in HBE cell line (Fig. 5B). We next perform double immunofluorescence staining of E-cadherin with vimentin of A549 cells, finding that E-cadherin is downregulated and vimentin is upregulated, which is consistent with WB results (Fig. 5D). Moreover, scratch test and invasion and migration test also confirmed that the migration ability of the A549 cells was enhanced (Fig. 5C, F). The above results indicate that OPN is able to induce epithelial cell EMT in vitro.

### OPN signals through CD44 to mediate alveolar epithelial cells EMT and fibrogenic protein expression

**Fig. 6:**
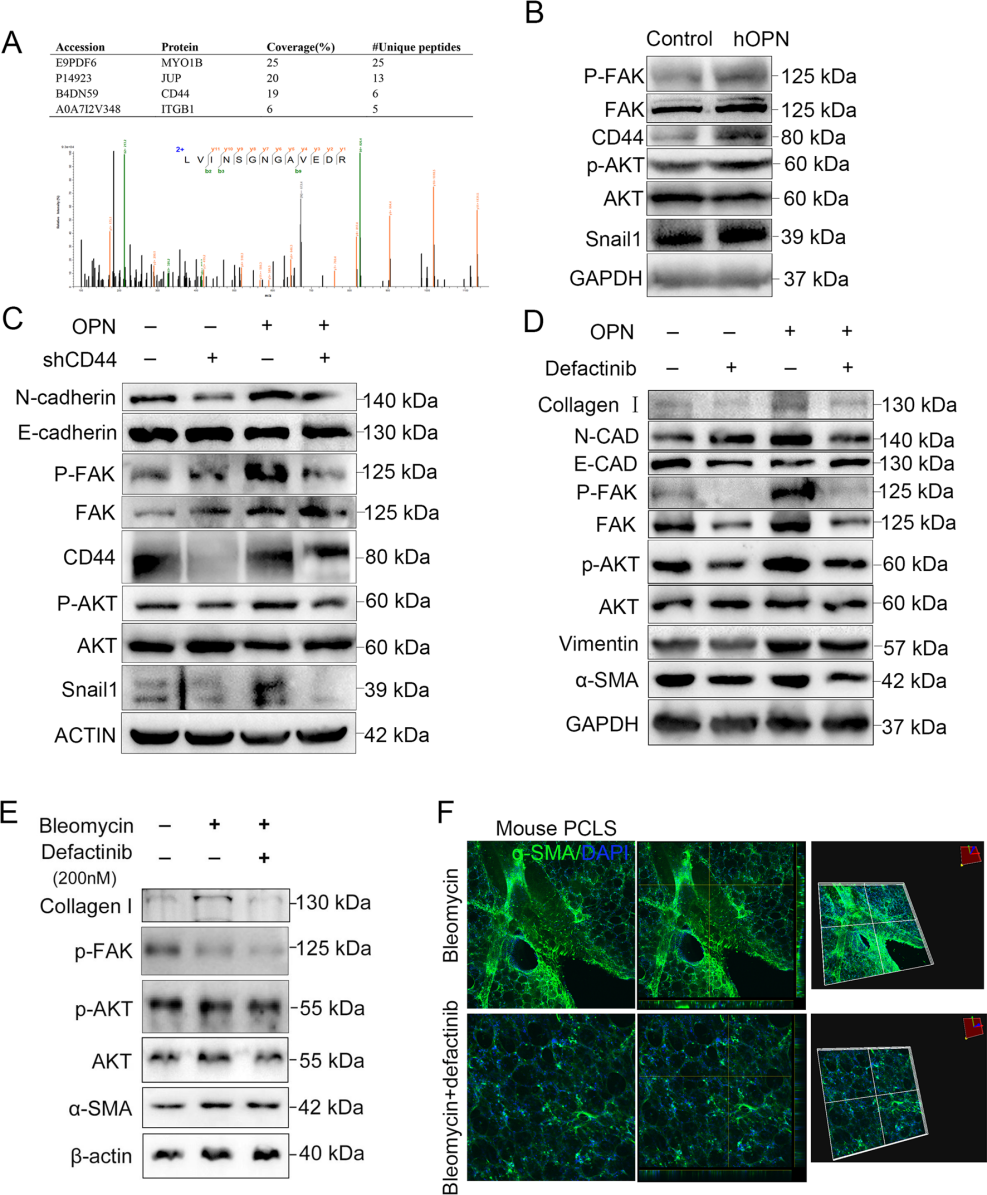
OPN drives fibrogenic protein synthesis via activation of the CD44/P-FAK/P-AKT cascade. **A**, **B** A549 cells were treated with OPN (1 μg/ml) or not, and harvested CO-IP test and western blot after 48 h. **A** Identification of CD44 from CCL1 immunoprecipitates in alveolar epithelial cells by MS analysis. **B** Immunoblots of CD44, P-FAK, FAK, P-AKT, AKT, Snail1 of A549 cells. **C** A549 cells were transfected with LV-negative control (NC) or LV-CD44-siRNA and then treated with OPN (1 μg/ml) for 72 h. Immunoblots of P-FAK, FAK, P-AKT, AKT, CD44, Snail1, N-CAD and E-CAD of A549 cells. **D** A549 cells were pretreated with defactinib and treated with OPN 1 h later for 48 h. Immunoblots of Collagen I, P-FAK, FAK, P-AKT, AKT, Vimentin, α-SMA, N-CAD and E-CAD of A549 cells. **D**, **E** PCLS were obtained from control mice and mice with pulmonary fibrosis and treated with P-FAK inhibitor. **E** Immunoblots of Collagen I, P-FAK, P-AKT, AKT, α-SMA of PCLS. **F** Confocal microscopy showed α-SMA (green), and DNA dye DAPI (cyan) in bleomycin PCLS treated P-FAK inhibitor with and matched group, showing fibrogenic protein synthesis

To explore the mechanism of OPN-induced EMT in alveolar epithelial cells, mass spectrometry was used to detect the proteins contained in the OPN-treated CO-IP protein mix samples that could bind to OPN-treated Co-IP protein. Mass spectrometry analysis showed that the proteins MYO1B, JUP, CD44 and ITGB1 were the most abundant in the OPN-CO-IP mixture (Fig. 6A). Among them, CD44 and ITGB1 are known downstream ligands of OPN, and CD44 is related to EMT of epithelial cells. Literature review found that CD44 and its downstream P-FAK/P-AKT pathway can be involved in the occurrence of epithelial EMT. Therefore, we examined the expression of this pathway in alveolar epithelial cells after OPN treatment. The results showed that after OPN treatment, P-FAK was increased in epithelial cells, its downstream pathways were activated, and the expression of epithelial cell markers was decreased, while the expression of mesenchymal cell markers was increased (Fig. 6B). To further clarify the role of CD44 in OPN-induced EMT in alveolar epithelial cells and in inducing downstream pathway activation, we used small interfering RNA to knock down CD44 in epithelial cells and then treated with OPN. The results demonstrated that knockdown of OPN effectively alleviates OPN-induced EMT and its downstream P-FAK/P-AKT/SNAIL1 signaling pathway (Fig. 6C). In order to further clarify the role of P-FAK, we further treated the epithelial cells with P-FAK inhibitor defactinib and found that inhibition of FAK phosphorylation inhibited its downstream pathways and ultimately attenuated OPN-induced EMT (Fig. 6D). The above results confirmed that OPN caused epithelial EMT through CD44/P-FAK/P-AKT signaling pathway. Further, we examined the effect of inhibition of FAK phosphorylation on pulmonary fibrosis using a mouse PCLS model. It was found that the expression of collagen and α-SMA in fibrotic PCLS was significantly inhibited after FAK phosphorylation inhibitor was used (Fig. 6E, F).

### Therapeutic targeting of OPN reduces PF

**Fig. 7:**
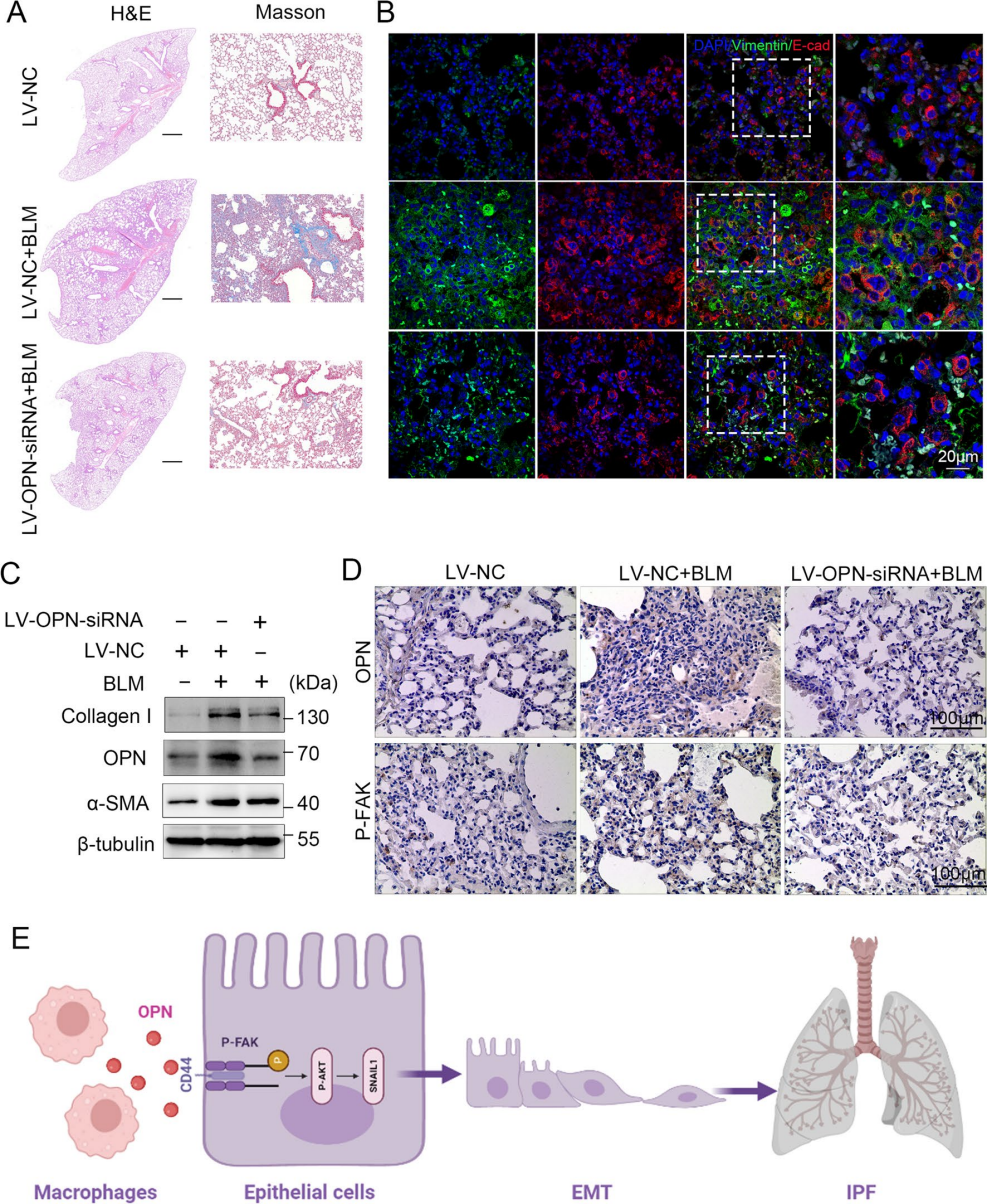
Silence of OPN attenuates epithelial-mesenchymal transition and pulmonary fibrosis. Mice (n = 10 in each group) were intratracheally injected with 1 × 109 TU LV-OPN-siRNA or negative control (NC) 7 days after administration of bleomycin. Mice were killed at day 21 after BLM instillation. **A** Pulmonary fibrosis was determined by hematoxylin–eosin (H&E) staining and Masson’s trichrome staining. Representative images of three independent experiments are shown. **B** Representative IHC OPN and P-FAK staining of mouse lung tissue. **C** Immunoblots of OPN, collagen and α-SMA of mouse lung tissue. **D** Confocal microscopy showing vimentin (green), E-cadherin (red), and DNA dye DAPI (cyan) in lung tissue section of mice, showing epithelial-mesenchymal transition. Bars, 100 µm. **E** Summary of the article

Based on the above results, we further explored the effect of OPN as a target to inhibit the occurrence of pulmonary fibrosis in mice. Considering that OPN is mainly expressed by macrophages, and macrophages are able to engulf foreign viruses, we employed a bleomycin-mediated lung fibrosis model in mice and administered LV-OPN-siRNA. The results of HE and MASSON staining showed that knockdown of OPN could effectively alleviate bleomycin induced pulmonary fibrosis in mice (Fig. 7A). Consistently, the expression level of OPN was reduced and the fibrotic protein collagen and α-SMA were also downregulated (Fig. 7B). Further immunohistochemistry and immunofluorescence staining showed that knockdown of OPN could effectively inhibit the expression of P-FAK and the oEMT in epithelial cells (Fig. 7C, D).

## Discussion

In this study, we found that OPN could promote the occurrence and development of IPF by acting on epithelial cells and promoting the occurrence of EMT. Moreover, the prognosis of IPF patients is related to the expression level of OPN, especially the content in bronchoalveolar lavage fluid. In detail, we demonstrated that OPN could induce EMT in epithelial cells and CD44/ p-FAK pathway played a key role in this process both in vivo and in vitro. Different from the conventional experimental methods, we established a PCLS model in the process of experimental design to verify the experimental conclusions more comprehensively. Finally, the OPN-knockdown mouse model further explored the effect and feasibility of targeting OPN to inhibit the progression of pulmonary fibrosis. This is a detailed exploration of the role of OPN in the pathogenesis and clinical application of pulmonary fibrosis (Fig. 7E).

Before this, the role of OPN in IPF has also been slightly mentioned in some studies. In 2020, Xianhua Gui found that elevated OPN could be a potential serum predictor for Acute exacerbation (AE) status and survival in IPF patients [16]. Besides, the level of OPN in peripheral blood was found to be associated with the survival rate of patients with IPF after anti-fibrosis treatment [23]. In addition, in our previous bioinformatics analysis, OPN was found to be a marker of IPF [24], which further verified the conclusions of other laboratories. The above findings collectively indicate that OPN plays an important role in the occurrence and development of IPF, which also lays a solid foundation for our study. However, considering the important role of OPN in other types of diseases such as cancer, we further explored the correlation between the expression level of OPN in bronchoalveolar lavage fluid and IPF. Compared with the expression level in peripheral blood, the expression level of the protein in bronchoalveolar lavage fluid can specifically reflect the expression of the protein in the lung, thereby excluding the interference of other organs. Because it is difficult to collect cases and follow up patients, we choose the meanalysis of the information in the database to obtain the information we want. In the present study, we first verified that the amount of OPN in bronchoalveolar lavage fluid was significantly higher in IPF than in controls. We further found that OPN in BALF was associated with the prognosis of IPF patients, and the correlation was higher than that in peripheral blood. These results suggest that the OPN content in BALF is more valuable than that in peripheral blood for the clinical application of IPF.

With the conclusions obtained from the human database, we further examined the relevant expression in the mouse model. Here, we took the classic mouse model of pulmonary fibrosis induced by a single tracheal instillation of bleomycin and collected samples at various stages for detection [25]. The results showed that the expression level of OPN was higher than that of the control group during all stages of pulmonary fibrosis in mice. Interestingly, OPN expression levels in BALF of mice were all maintained at high levels, while OPN expression levels in peripheral blood were significantly different only at early stages. This result is similar to the results of human database. We speculate that with the development of pulmonary fibrosis, the structure of lung tissue is destroyed and the alveolar collapse loses the function of material exchange with blood vessels, which leads to the accumulation of OPN in the lung and cannot enter the peripheral blood. This may also be why the results of bronchoalveolar lavage fluid are more valuable for clinical diagnosis and prediction of patient prognosis.

In recent years, epithelial cell damage has been identified as a driver cell in the development of pulmonary fibrosis. In addition to their gas and substance exchange functions, epithelial cells are also barrier cells of the lung [7]. Under a variety of stimuli, epithelial cells will undergo EMT, senescence, apoptosis and other phenomena [6]. However, in our in vitro pilot study, we found that OPN did not induce epithelial cell senescence and cell death. Since the 1980s, EMT has been recognized as one of the key mechanisms involved in fibrosis in IPF [26]. In addition, in cancer-related studies, EMT also contributes to the invasion and migration of cancer cells and accelerates the course of cancer [27]. However, it is slightly different from the progression of cancer, in the development of pulmonary fibrosis, when the alveolar epithelial cells are EMT, they are often accompanied by the transformation into fibroblasts [5, 28]. In our study, the expression of fibrosis-related proteins was similarly found to be upregulated after OPN treatment.

In our study, multiple models were also used to verify individual experimental hypotheses. Firstly, in order to explore whether OPN can cause pulmonary fibrosis in mice and exclude the interference of other pro-fibrotic factors, we established an animal model by multiple tracheal instillation of OPN. The inspiration came from an article exploring the profibrotic role of CCL1 [29]. Secondly, to explore the effect of OPN downstream pathway on lung fibrosis, we used pathway inhibitors to treat PCLS [30], so that the structure and fibrosis degree could be directly detected at the tissue level. Finally, we also established OPN knockdown mice and further explored the effect of OPN as a therapeutic target using bleomycin treatment.

In summary, we found that OPN secreted by macrophages can act on alveolar epithelial cells, bind to CD44 on their membrane, and further phosphorylate FAK, thereby promoting the phosphorylation of AKT and the expression of downstream EMT-related proteins, and promote the occurrence of pulmonary fibrosis. The results of this study are helpful to further explore the pathogenesis of pulmonary fibrosis, propose new therapeutic targets and lay the foundation for further research.

### Supplementary Information

**Additional file 1: Figure S1.** Additional information about database related results. **A** Genes with p-value < 0.05 and logFC (> 2 or < − 2) in the volcano figure in Fig. 1A, GSE70866. **B** ROC curves for forecasting overall survival in GSE28042. **C** Kaplan–Meier plot of overall survival between OPN high and OPN low groups in GSE28042.

**Additional file 2: Figure S2. A**–**D** Negative control of immunofluorescence test. **A** Confocal microscopy showing mouse IgG (green), rabbit IgG (red), and DNA dye DAPI (cyan) in lung tissue section of IPF and mice, negative control of Fig. 3. **B** Confocal microscopy showing mouse IgG (green), rabbit IgG (red), and DNA dye DAPI (cyan) in biotin-OPN treated mouse lung tissue, negative control of Fig. 4I. **C** Confocal microscopy showing mouse IgG (green), rabbit IgG (red), and DNA dye DAPI (cyan) in OPN treated mouse lung tissue, negative control of Fig. 4J. **D** Confocal microscopy showing mouse IgG (green), rabbit IgG (red), and DNA dye DAPI (cyan) in A549 cells, negative control of Fig. 5D. **E** A549 cells were treated with OPN (1 μg/ml) or not for 48 h. Immunoblot of TGF-β of A549 cells. **F** Bleomycin (5 mg/kg body weight) or saline were administered daily in mice, and parameters analyzed on day 14. Recombinant mouse OPN in 50 µl of PBS (50 mg/kg) was instilled into the trachea every 3 days for one month. Immunoblots of OPN, α-SMA of the above mouse lung tissue. **G** Secondary mass spectrum of Fig. 6A.

**Additional file 3: Table S1.** Specifications of primary antibodies.

**Additional file 4: Table S2.** List of primers used for reverse transcription-quantitative polymerase chain reaction analysis.

## Data Availability

The data that support the findings of this study are available from the corresponding author upon reasonable request.
