# Supplementary figures and images for: Increased expression of OPN contributes to idiopathic pulmonary fibrosis and indicates a poor prognosis
Source: J Transl Med. 2023 Sep 19;21:640. doi: 10.1186/s12967-023-04279-0 (PMC10510122; doi:10.1186/s12967-023-04279-0)

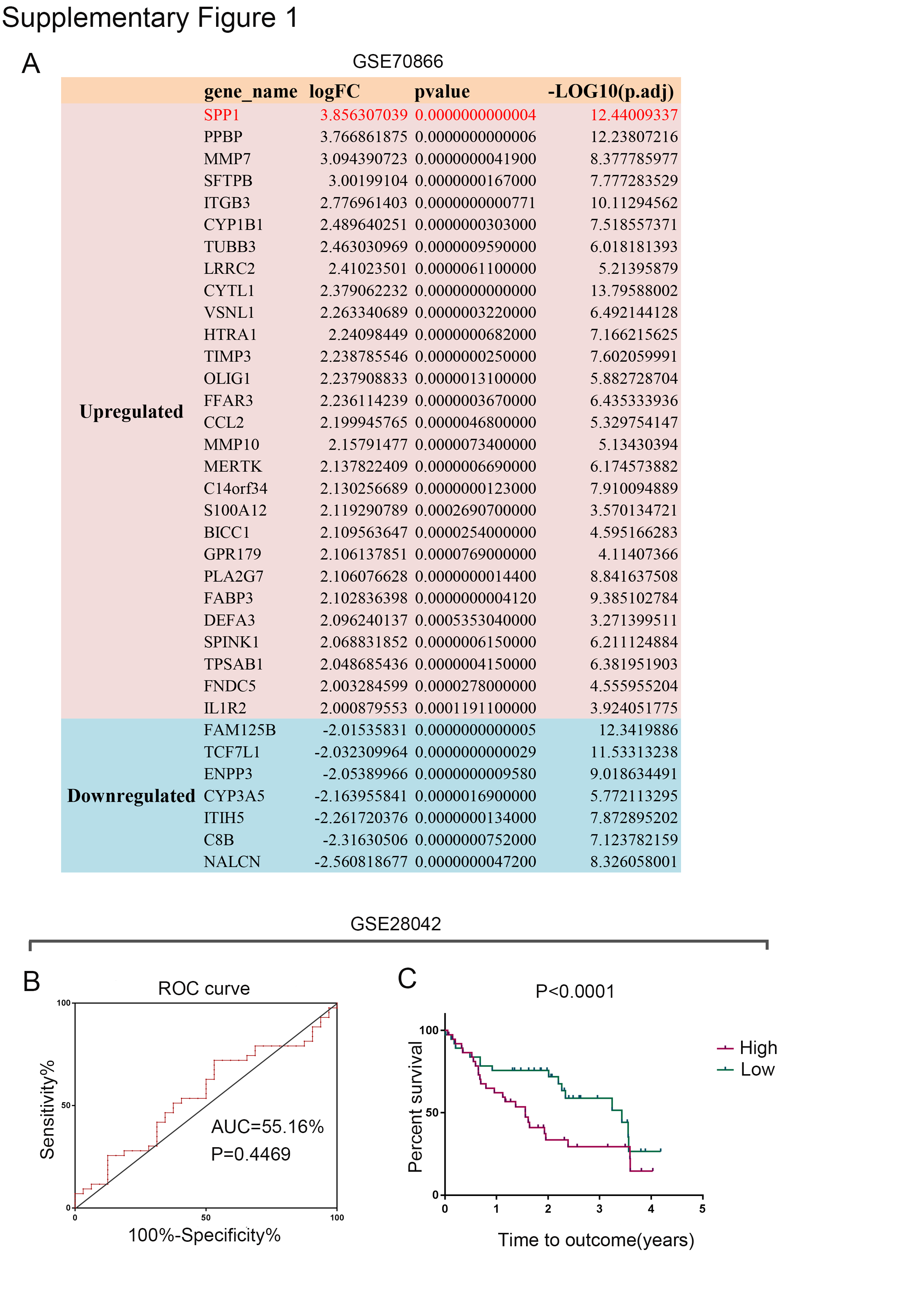

Supplement: Supplementary file 1 — Additional file 1: Figure S1. Additional information about database related results. A Genes with p-value < 0.05 and logFC (> 2 or < − 2) in the volcano figure in Fig. 1A, GSE70866. B ROC curves for forecasting overall survival in GSE28042. C Kaplan–Meier plot of overall survival between OPN high and OPN low groups in GSE28042. [file 12967_2023_4279_MOESM1_ESM.tif]

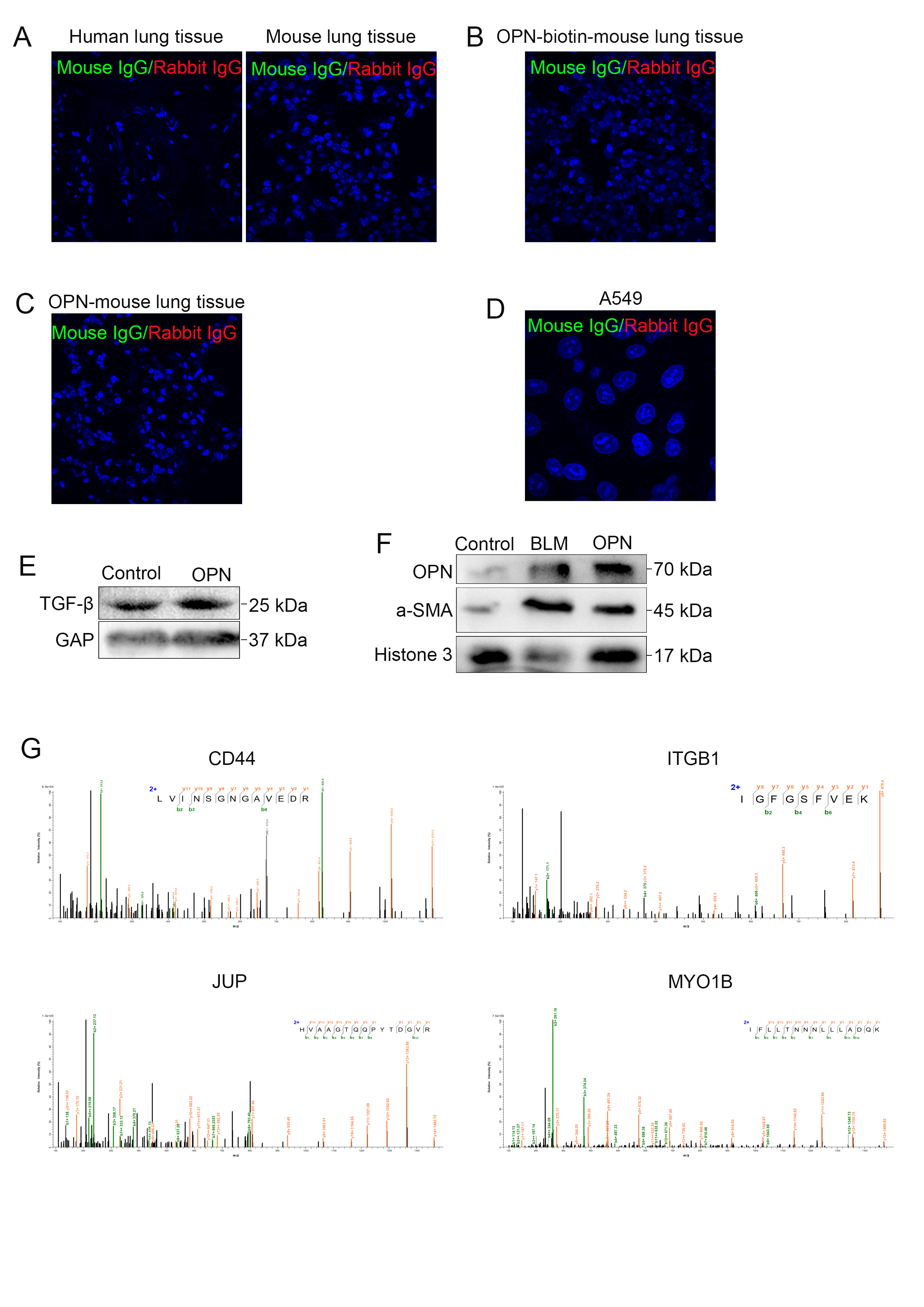

Supplement: Supplementary file 2 — Additional file 2: Figure S2. A–D Negative control of immunofluorescence test. A Confocal microscopy showing mouse IgG (green), rabbit IgG (red), and DNA dye DAPI (cyan) in lung tissue section of IPF and mice, negative control of Fig. 3. B Confocal microscopy showing mouse IgG (green), rabbit IgG (red), and DNA dye DAPI (cyan) in biotin-OPN treated mouse lung tissue, negative control of Fig. 4I. C Confocal microscopy showing mouse IgG (green), rabbit IgG (red), and DNA dye DAPI (cyan) in OPN treated mouse lung tissue, negative control of Fig. 4J. D Confocal microscopy showing mouse IgG (green), rabbit IgG (red), and DNA dye DAPI (cyan) in A549 cells, negative control of Fig. 5D. E A549 cells were treated with OPN (1 μg/ml) or not for 48 h. Immunoblot of TGF-β of A549 cells. F Bleomycin (5 mg/kg body weight) or saline were administered daily in mice, and parameters analyzed on day 14. Recombinant mouse OPN in 50 µl of PBS (50 mg/kg) was instilled into the trachea every 3 days for one month. Immunoblots of OPN, α-SMA of the above mouse lung tissue. G Secondary mass spectrum of Fig. 6A. [file 12967_2023_4279_MOESM2_ESM.tif]
