# Supplementary material for: Increased expression of OPN contributes to idiopathic pulmonary fibrosis and indicates a poor prognosis
Source: J Transl Med. 2023 Sep 19;21:640. doi: 10.1186/s12967-023-04279-0 (PMC10510122; doi:10.1186/s12967-023-04279-0)
Supplement: Supplementary file 3 — Additional file 3: Table S1. Specifications of primary antibodies. [file 12967_2023_4279_MOESM3_ESM.docx]

Additional file 3: Table S1. Specifications of primary antibodies

| **Antibody** | **Species** | **Company** | **Catalog** | **Dillution** |
| --- | --- | --- | --- | --- |
| Anti-OPN | Rabbit polyclonal antibody | Protentech | 22952-1-AP | 1:1000 (WB)  1:200 (IF)  1:400 (IHC) |
| Anti-F4/80 | Rabbit polyclonal antibody | Protentech | 29414-1-AP | 1:200 (IF) |
| Anti-E-cadherin | Rabbit polyclonal antibody | Protentech | 20874-1-AP | 1:1000 (WB)  1:200 (IF) |
| Anti-N-cadherin | Rabbit polyclonal antibody | Protentech | 22018-1-AP | 1:1000 (WB)  1:200 (IF) |
| Anti-Vimentin | Rabbit polyclonal antibody | Protentech | 10366-1-AP | 1:1000 (WB)  1:200 (IF) |
| Anti-Snail1 | Rabbit polyclonal antibody | Protentech | 13099-1-AP | 1:1000 (WB) |
| Anti-P-FAK | Rabbit mAb | Abcam | ab81298 | 1:1000 (WB)  1:400 (IHC) |
| Anti-P-AKT | Mouse mAb | Protentech | 66444-1-AP | 1:1000 (WB) |
| Anti-FAK | Mouse mAb | Protentech | 66258-1-AP | 1:1000 (WB) |
| Anti-AKT | Mouse mAb | Protentech | 60203-1-AP | 1:1000 (WB) |
| Anti-α-SMA | Mouse mAb | Boster | BM0002 | 1:1000 (WB)  1:200 (IF)  1:200 (IHC) |
| Anti-COL1A | Rabbit polyclonal antibody | Boster | BA0325 | 1:1000 (WB)  1:200 (IF) |
| Anti-β-catenin | Rabbit polyclonal antibody | abcam | ab6302 | 1:1000 (WB)  1:200 (IF) |
| Anti-ABCG2 | Rabbit mAb | abcam | EPR20080 | 1:200 (IF) |
| Anti-GAPDH | Mouse mAb | Absci | #AB40493 | 1:1000（WB） |
