# Supplementary material for: Increased expression of OPN contributes to idiopathic pulmonary fibrosis and indicates a poor prognosis
Source: J Transl Med. 2023 Sep 19;21:640. doi: 10.1186/s12967-023-04279-0 (PMC10510122; doi:10.1186/s12967-023-04279-0)
Supplement: Supplementary file 4 — Additional file 4: Table S2. List of primers used for reverse transcription-quantitative polymerase chain reaction analysis. [file 12967_2023_4279_MOESM4_ESM.docx]

| Gene | Primer sequence |
| --- | --- |
| SPP1 | F 5’-CGTGAGTCCCATTAAGATGGAGT-3’  R 5’-CCCGACAGTGGATATAGAACAGA-3’ |
| CDH1 | F 5’-GAAATGCCACCTTTTGACAGTG-3’  R 5’-TGGATGCTCTCATCAGGACAG-3’ |
| CDH2 | F 5’-TGGATGCTCTGTACGGGAAG-3’  R 5’-CCAGGCTGGTGTGAAACTGAA-3’ |
| Vimentin | F 5’-TTTCCGCAAGGTTCGATTTTCA-3’  R 5’-GGCATCTGCGCTCTACCATC-3’ |
| 18S | F 5’-ACCTGACCTGCCGTCTAGAA-3’  R 5’-TCCACCACCCTGTTGCTGTA-3’ |

Additional file 4: Table S2. List of primers used for reverse transcription-quantitative polymerase chain reaction analysis.
